# *E. coli* Heat-labile Enterotoxin B Subunit as a Platform for the Delivery of HIV Gag p24 Antigen

**DOI:** 10.4172/2155-9899.1000140

**Published:** 2013-04-23

**Authors:** Karmarcha Martin, Toufic O Nashar

**Affiliations:** College of Veterinary Medicine, Nursing and Allied Health, Department of Pathobiology, Tuskegee University, Tuskegee, AL 36088, USA

**Keywords:** HIV, Gag p24, Vaccines, Enterotoxins

## Abstract

Multiple vaccination strategies have been devised against HIV-1 including delivery of HIV moieties in attenuated or replication defective recombinant microbial agents alone or in combination with priming agents in form of soluble proteins or naked DNA. For the priming agents to be effective, adjuvants might be essential in directing the immune response to a desired outcome. *E. coli* enterotoxin B subunit (LTB) is an effective adjuvant and carrier for other proteins and epitopes. Here we show that conjugation of HIV gag p24 to LTB enhances the T cell response to gag p24 by increasing rate of T cell division compared to other treatments. Because HIV vaccines are likely to be multivalent, we further investigated whether gag p24 inhibits antigen presentation of an unrelated antigen, OVA. Addition of gag p24 to OVA-responsive DO.11.10 cell culture did not have adverse effects on antigen presentation. Interestingly, the presence of LTB in these cultures significantly increased proliferation of DO.11.10 cells. In all, the results suggest the use of LTB to boost immune responses against HIV gag p24 in systemic priming regimens with oral recombinant HIV vaccines.

## Introduction

A number of different strategies are sought in the fight against the Human Immunodeficiency Virus- 1 (HIV-1). Most of these are directed to deliver and stimulate immune responses to HIV moieties expressed by recombinant viruses or bacteria [1–5]. For example, we have recently shown that recombinant coxsakievirus expressing a 73 aa sequence of HIV gag p24, induced high level of T cell responses [6]. Additional strategies involve systemic priming followed by oral delivery of the vaccine. Among the systemic priming agents are DNA expressing HIV proteins, HIV gag p24 targeted by antibody to antigen presenting cells (APC), and HIV gag p24-Fc fusion protein [1,7,8]. Thus, a number of proteins could form the basis of vaccination against HIV-1. The most studied are two envelope proteins: surface glycoprotein gp120 and transmembrane glycoprotein gp 41 involved in attachment and entry of the virus into the host cell [9]. Other important proteins form various structures in the virus and are grouped under the name “group-specific antigens” (Gag). The HIV-1 gag proteins are formed initially as a polyprotein precursor Pr55 gag (of 55 KD) which is cleaved off after virus budding into mature protein p17 (matrix), p24 (capsid), p7 (nuclocapsid), and p6 [9,10]. Gag p24 form the viral capsid that mediates protection of viral RNA. Gag p24 also plays an important role in virus assembly and maturation inside the host cell and before budding out of the membrane [9]. Moreover, gag p24 has been shown to be fairly conserved among HIV-1 isolates [11,12]. HIV-1 gag protein has also been shown to be one of the major CTL antigens in HIV positive individuals [13–15]. Moreover, absence of antibodies to HIV gag early or late after infection correlates with poor prognosis of HIV-1 infected individuals [16,17]. Hence, humoral and cellular responses to HIV gag appear to play a vital role in limiting progression of the disease.

For HIV proteins to be effective in priming regimens, immunomodulating agents are sought to act as adjuvants to boost and direct the immune response to a desired outcome. Such adjuvants ought to act locally by increasing availability of mature antigen presenting cells (APCs) in the appropriate compartment, modulating B and T cell immune responses to foreign epitopes by acting directly on the APCs, and focusing immune responses to critical epitopes. We previously demonstrated that the B subunits of *Escherichia coli* enterotoxin (LTB) increase expression of stimulatory and accessory proteins on APCs, including, MHC class II, B7-2, CD25, and ICAM-1, and result in increased CD4^+^ T-cell activation [18–21]. Further, when ovalbumin (OVA) was conjugated directly to LTB and targeted to APCs, the T cell response to OVA was significantly enhanced relative to OVA taken up by fluid-phase endocytosis [20]. Several mechanisms could explain these findings, including, high affinity binding of LTB to its cell surface receptor ganglioside G_M1_ [18–20] resulting in enhanced targeting and access to MHC compartments [20,22], increased activation of APCs and T cells [19,21], and enhanced stability of the conjugated antigens. Thus, LTB and the closely related cholera toxin B subunits are excellent carriers for other antigens and epitopes [18,23–26].

Here we conjugated HIV gag p24 to LTB and analyzed the T cell response to gag p24. Because HIV gag p24, and other HIV proteins, have been reported to inhibit the immune response and antigen presentation to an unrelated antigen [27,28], we also sought to look at effects of adding the protein in DO.11.10 T cells culture in the absence and presence of LTB. We show that conjugation of HIV gag p24 to LTB enhances the T cell response by increasing rate of cell division compared to other treatments. Further, although addition of gag p24 to DO.11.10 culture did not have adverse effects on antigen presentation, the presence of LTB significantly increased their division rate. In all, the results suggest the use of LTB and HIV gag p24 in prime/boost regimens with oral recombinant HIV vaccines. This is the first report that uses LTB as a platform to evaluate the immune response to HIV gag p24.

## Materials and Methods

### Animals

Female BALB/c mice of 8 weeks old were purchased from Harlan Olac, USA. Animals were housed at Tuskegee University animal facility. The animal protocol has been approved by Tuskegee University Bio-safety Committee.

### Reagents

Heart infusion powder (Sigma, St. Louis, MO) was used to grow *E. coli* BL-21. Clontech purification kit and Histalon metal affinity columns (Clonetch, Mountain View, CA) were used to lyse *E. coli* bacteria and purify gag p24. Recombinant *E. coli* heat-labile enterotoxin B subunits expressed in the yeast *Pichia pastoris* (Sigma) was used. The bi-functional crosslinker Succinimidyl 3-(2-pyridyldithio) propionate (SPDP) (EMD Millipore, Billerica, MA) was used. Ganglisoside GM1 receptor was from Sigma. Mouse HIV-1 p24 antibody (#Ant-152-b, Prospec, Israel) and goat anti-mouse alkaline phosphatase conjugate (Sigma) were used. Hanks Balanced Salt Solution (HBSS) and RPMI 1640 were from Lonza, USA. Dulbecco’s Modified Eagle’s Medium (MDEM) was from Thermo Scientific, Rockford, IL. Addititives to culture medium included, Penicillin/Streptomycin (Corning, USA); L-glutamine (Sigma); Hepes (Thermo Scientific) and sodium pyruvate (Thermo Scientific). Fetal bovine serum (Thermo Scientific) was used as a supplement to culture medium. Bovine serum albumin and ovalbumin were both from Sigma. CellTrace proliferation kit (CFSE, Life Tech., USA). DO.11.10 hybridoma was a kind gift from *Prof. Jim Drake*, Albany Medical College, NY. Recombinant *E. coli* BL-21 expressing His-HIV gag p24 was a kind gift from *Prof. Venigalla Rao,* Catholic University, Washington DC.

### Growth and expression of HIV gag p24 in *E. coli* BL-21

*E. coli* competent BL-21 was grown from a start-up culture in the presence of Ampicillin and Chloramphenicol at 30°C, overnight on a shaker-incubator. The next day 1 mM of Isopropyl β-D-1-thiogalactopyranoside (IPTG) was added to each flask at optical density between 0.4 and 0.5, for the last three hours. The bacterial culture was spun down for 20 min, 4°C, 10,000 rpm. The supernatant was removed and the pellet weighed. 2 ml of extraction buffer (Histalon purification kit) per 100 mg of pellet was added to the pellet, gently mixed and left on ice for 15 minutes with intermittent mixing. The lysate was cleared from the bacterial membranes by centrifugation at 4°C for 20 minutes. The supernatant containing His-gag p24 protein was carefully collected.

### Purification of HIV gag p24

Purification of gag p24 was based on the use of Histalon columns containing metal affinity resin that binds with high affinity and specificity to histidine (His)-tagged proteins. The column was equilibrated with 5–10 ml of equilibrium buffer (Histalon purification kit). The bacterial lysate sample was loaded into the column. 1 ml fractions were collected for subsequent gel analysis. The column was washed with 8 ml of equilibrium buffer followed by 7 ml of wash buffer. Gag p24 was eluted from the column with 5–8 ml of elution buffer. 1 ml fractions were collected. The presence of gag p24 in the fractions was monitored by measuring optical density at 280 nm, and by SDS-PAGE gel analysis. Protein concentration was estimated by absorbance at 280 nm with an exclusion coefficient of 10.

### Conjugation of LTB and HIV gag p24

The conjugation was performed using the bi-functional cross-linker N-succinimidyl 3-(2-pyridydithio) propionate (SPDP). SPDP was equilibrated at room temperature. 2 mg of SPDP was dissolved in 320 μl of DMSO. 200 μg of commercial LTB was dissolved in 1 ml of Phosphate Buffer Saline (PBS) pH 7.4 and dialyzed against PBS. 25 μl of the SPDP solution was added to LTB. The mixture was dialyzed twice against PBS pH 7.4. In this way, LTB was activated by the binding of SPDP to its amino group. Sulfhydryl-containing purified Gag p24 was then added to the SPDP-LTB reaction and left overnight at room temperature. Thus, the conjugation of the two proteins occurred via a disulfide bond. The conjugate was subsequently dialyzed against PBS pH 7.4 and analyzed by SDS-PAGE and ELISA.

### Analysis of LTB-Gag p24 conjugate by G_M1_-ELISA

An ELISA method was developed for the analysis of LTB-gag p24 conjugate. The method is based on the high affinity binding of LTB to its receptor G_M1_. 5 μg/ml of G_M1_ in PBS pH 7.4 was coated to wells of a 96-well ELISA plate. The plate was incubated overnight at room temperature. To generate gag p24 positive control, other wells were coated with 40 μg/ml of purified gp24 in PBS for 1 hr at room temperature, followed by washing the excess protein. Following washing with PBS, all wells were blocked with 200 μl of 1% BSA in PBS and the plate was incubated at 37°C for 1 hour. This was followed by 100 μl/well of the conjugated mixture of LTB-gag p24 serially diluted (2×) in PBS/0.1%BSA. The wells coated with gag p24 were filled with PBS only. The plate was incubated for 1 hour at room temperature. Following washing with PBS/0.1% BSA/0.05% Tween 20, mouse anti-gag p24 1/10,000 diluted in PBS/0.1% BSA/Tween 20 was added to all wells. The plate was incubated at room temperature for 2 hours. This antibody was omitted in negative control wells. Following washing, goat anti-mouse alkaline phosphatase conjugate diluted at 1/25,000 in PBS/0.1% BSA/Tween 20 was added and incubated overnight at 4°C. Following washing, wells were incubated with alkaline phosphatase substrate for 30 minutes after which the development of color was read in a spectrophotometer at 405 nm.

### Immunization of mice

Four groups consisting of 3 mice each were injected each with 40 μg LTB-p24 conjugate; 40 μg LTB+40 μg gag p24; 40 μg gag p24 protein alone, or PBS. All injections were given by the intraperitoneal (i.p) route in 200 μl PBS. The mice were fed and watered normally for 13 days after which they were euthanized.

### Isolation of cells from the spleen and mesenteric lymph nodes (MLNs)

The spleen and mesenteric lymph nodes (MLNs) were collected from each individual animal and pooled for each group. These provided a source of APCs (spleen, ~65%) and a source of T cells (MLNs, ~90%). Isolation of cell suspension was made by placing each organ from each animal in each group in 0.2 μm cell filters immersed in HBSS. 3 spleens or 3 MLNs were homogenized separately with the back of a sterile syringe. The filtrate is then taken and loaded in 50 ml Falcon tubes and centrifuged at 1500 rpm. Supernatants were carefully removed and the cell pellet was washed 2× in HBSS, 1 final in “Complete RPMI” (containing 2 mM L-glutamine, 50 μM of 2-mercaptoethanol, 100 units/ml penicillin+100 μg/ml streptomycin sulfate, 20 mM Hepes).

### Antigen presentation assay for the measurement of the T cell response to gag p24 in mice

Splenic cells at 2×10^6^/ml, 2 ml/well were incubated with 50 μg/ml of gag p24 for 5 hrs at 37°C in 5% CO_2_ to allow uptake by the APCs. The cells were cultured in “Complete RPMI” medium supplemented with 0.5% of fresh mouse autologous serum. The MLNs cells were pre-labeled with a predetermined optimal concentration of 5 μM CFSE. MLN cells were incubated at 37°C in 5% CO_2_ at 2×10^6^/ml for 3 hours to deplete adherent cells. Supernatants were then carefully transferred in equal volume to wells containing the splenic APCs, resulting in a final concentration of 1×10^6^/ml cells/well. The cells were then incubated for 4 days. After incubation, the cells were spun down, washed once by centrifugation, resuspended in PBS+0.1% BSA, and analyzed by flow cytometry (Becton Dickenson). The FlowJo software (Tree Star, OR) was used for acquisition and analysis of cell proliferation.

### Antigen presentation assay for the measurement of the effects of gag p24 and LTB on T cell division of DO.11.10

Splenic cells were isolated from normal mice and pooled. The cells were incubated in 96-well tissue culture plates in the absence of antigen or with OVA (0.5 mg/ml); OVA (0.5 mg/ml)+gag p24 (10, 20 and 40 μg/ml); OVA (0.5 mg/ml)+gag p24 (10, 20 and 40 μg/ml)+30 μg/ml LTB; and OVA (0.5 mg/ml)+LTB (30 μg/ml), for 6 hrs at 37°C to allow uptake. All proteins were dialyzed against PBS pH 7.4 and filter sterilized before addition to culture. The cells were cultured in MDEM containing 2 mM L-glutamine, 50 μM 2-mercaptoethanol, 100 units/ml penicillin+100 μg/ml streptomycin sulfate, 1mM sodium pyruvate, 20 mM Hepes, and 10% fetal bovine serum. DO.11.10 T cells were grown in MDEM, washed and pre-labeled with CFSE as detailed above. They were subsequently added to splenic cells at a ratio of 1×10^5^ splenic cells to 2.5×10^4^ T cells per 100 μl total volumes. 5 replicate wells were included for each treatment. The cells were then incubated for 3 days and analyzed by flow cytometry.

## Results

### Chemical conjugation of LTB and HIV gag p24

**Figure 1:**
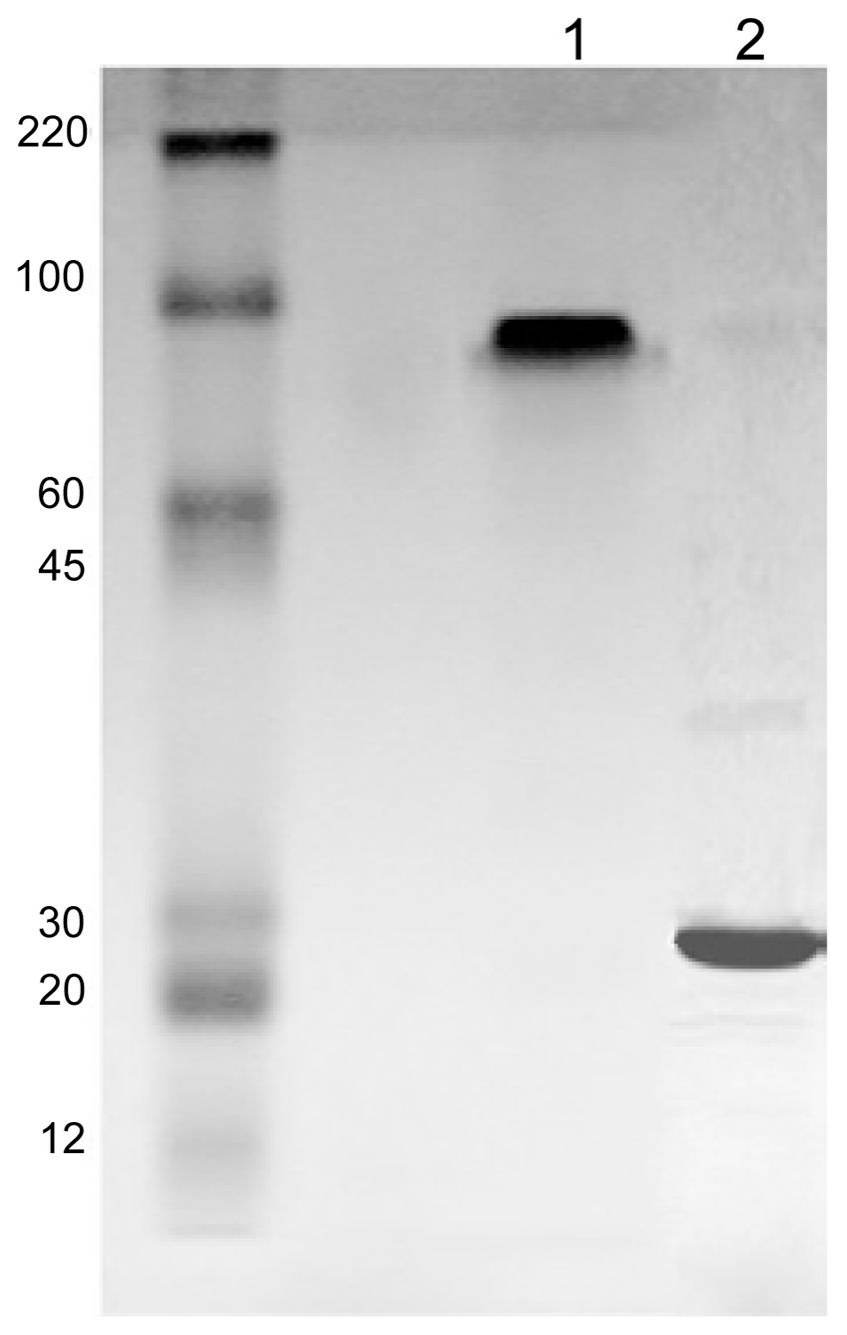
Purification of HIV gag p24 *E. coli* competent BL-21 containing His-gag p24 plasmid was grown overnight and induced by IPTG. The bacterial culture was spun down and the pellet lysed with extraction buffer (Histalon purification kit). The lysate was cleared by centrifugation and the supernatant was loaded into Histalon metal affinity column. Following washing, the column was loaded with elution buffer. The eluate was then collected, dialyzed against PBS, pH 7.4, and analyzed on 10% protein gel. Molecular weight marker was also included. The gel shows highly pure protein (gag p24) of approximately 78 KD in non-reduced form and 26 KD in the reduced form.

Figure 1 shows gel analysis of purified HIV gag p24. In non-reduced form, the protein runs at 78 KD in SDS-PAGE whereas in reduced form it runs at 26 KD. The purified protein is highly pure (estimate>95%).

**Figure 2:**
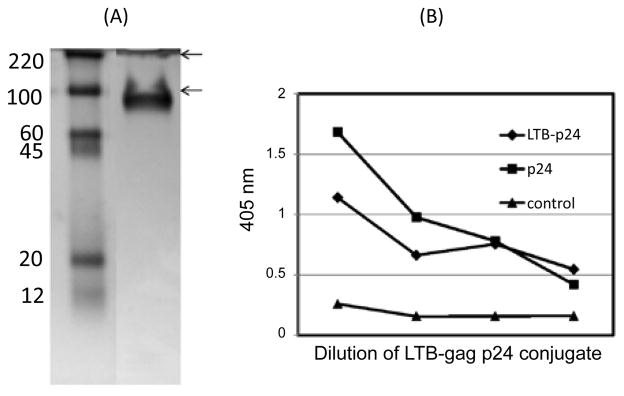
Analysis of the LTB-HIV gag p24 conjugate Conjugation of HIV gag p24 to LTB was performed by using the bi-functional crosslinker, SPDP. (A) The conjugate was dialyzed against PBS pH 7.4, loaded and run on 10% protein gel under non-reducing condition. The gel shows 2 bands, a lower band of approximately 100 KD and a higher band on the top of the gel of approximately 200 KD. (B) A GM1-ELISA was performed for the analysis of the conjugate. The conjugate was probed on GM1-coated wells. The conjugate was serially diluted (2×) and probed with mouse anti-gag p24 and goat anti-mouse alkaline phosphatase. The positive control consisted of mouse anti-gag p24 and goat anti-mouse alkaline phosphatase incubated in wells coated with gag p24 (p24 control). The negative control consisted of GM1 wells incubated with LTB-gag p24 conjugate and goat anti-mouse alkaline phosphatase in the absence of the primary antibody. The reaction was developed by addition of alkaline phosphatase substrate and read at 405 nm.

SPDP is a bi-functional crosslinker that reacts with an amino group of one protein and provides a thiol group that reacts with a corresponding thiol group in the other protein thus forming a stable disulfide bond. Each monomer of LTB and gag p24 contains 2 thiol groups. LTB is a pentamer and gag p24 is a trimer thus providing 10 and 6 thiol groups, respectively. Initial experiments that involved reacting the thiol groups of SPDP and LTB did not result in an optimum conjugate because a significant amount of LTB was unconjuagted. However, a similar reaction of SPDP with HIV gag p24 did result in a stable conjugate as shown in Figure 2A. The conjugate ran at 100 KD. A fainter band appeared at 200 KD. All the proteins in the mixture appear to have been conjugated as these were not detected. To further analyze the conjugate, a G_M1_-ELISA was performed. Probing with anti-gag p24 antibody confirmed the presence and stability of the LTB-gag p24 conjugate (Figure 2B).

### Immunization of mice with LTB-gag p24 conjugate enhances the HIV gag p24-specific T cell response

Having produced a stable LTB-gag p24 conjugate, it was important to prove that the conjugation to LTB results in enhanced T cell responses compared to non-conjugated gag p24. An enhancement of the anti-gag p24 T cell response by the LTB-gag p24 conjugate would be expected as a result of LTB binding to the G_M1_ receptor on surface of antigen presenting cells. This was compared to a mixture of free LTB and free gag p24 to see if the conjugation of gag p24 to LTB offers any advantage.

Mice were immunized with the conjugate, a mixture of LTB and gag p24, gag p24 alone or PBS. All proteins were injected once and in PBS. 13 days after immunization, splenic cells were isolated from each group pooled and incubated with purified gag p24 for 5 hrs. MLNs cells were collected, pooled and pre-labeled with CFSE before they were added to the splenic cultures. Cells were then analyzed by flow cytometry after 4 days.

**Figure 3:**
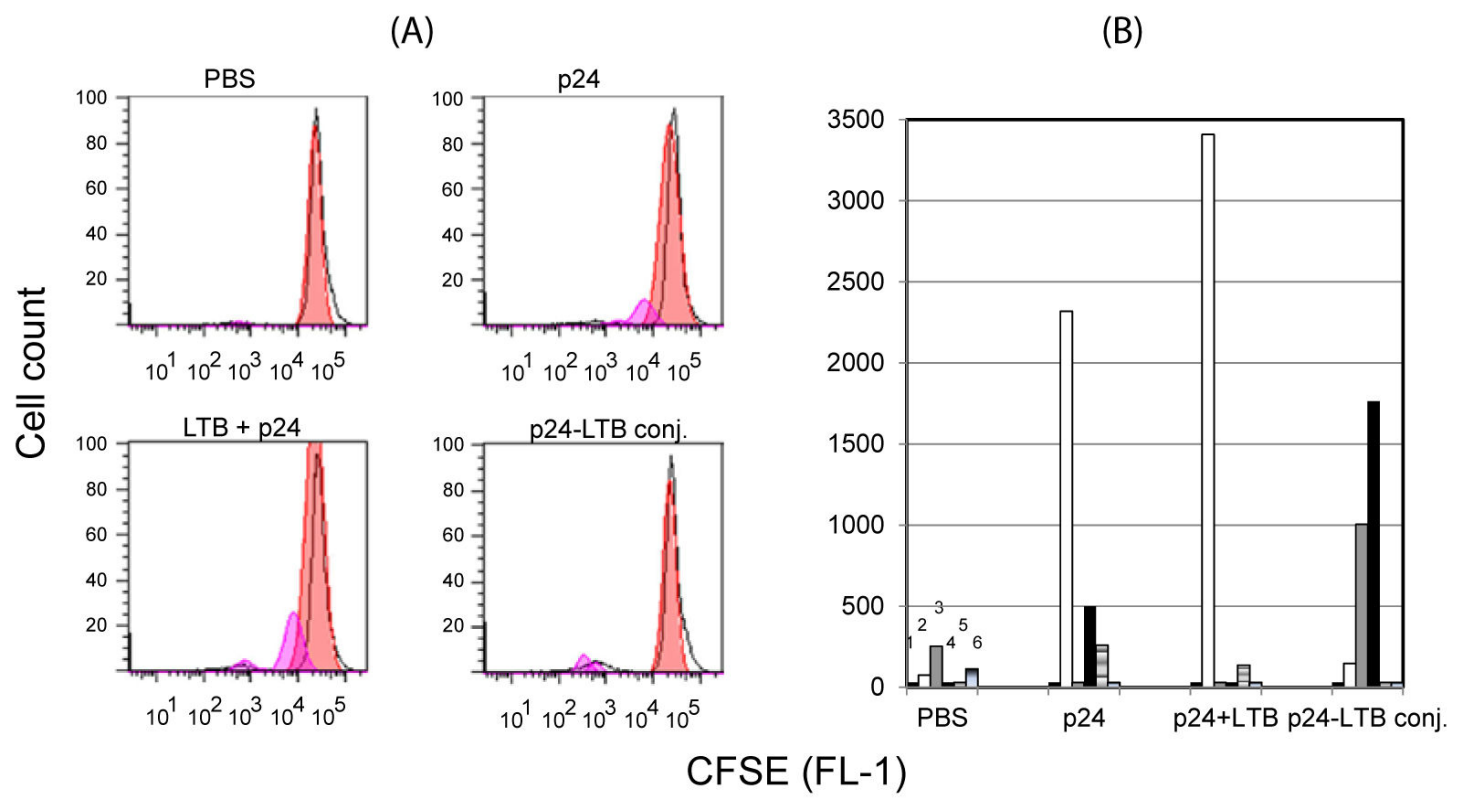
LTB-gag p24 conjugate enhances the T cell response to HIV gag p24 Groups of mice were injected i.p. with 40 μg LTB-p24 conjugate; 40 μg LTB+40 μg gag p24; 40 μg gag p24 protein alone, or PBS. 13 days after initial immunization, splenic cells (as a source of APCs, 65%) were isolated from these mice, pooled and incubated with gag p24 for 5 hrs. Mesenteric lymph nodes (as a source of T cells, 90%) were pre-labeled with CFSE and added. Cultures were incubated for 4 days. On day 4, cells were harvested, washed, and acquired by flow cytometry. Data were analyzed by FlowJo software. Data shown represent pattern of cell division and numbers of divided cells (A and B). In B, the same data as in A is plotted to show more clearly the increase in cell division rate by the conjugate. 1, 2, 3, 4, 5, 6 represent generation numbers.

In the culture medium from the PBS group (Figure 3A), little or no cell division was detected. Cells isolated from mice immunized with gag p24 alone show mainly one major peak representing 1 division (generation 1). Smaller peaks were also detected representing generation 3 and 4. In comparison, cells isolated from mice immunized with the mixture of LTB+gag p24 showed one major peak in generation 1 with no major peaks detected further, an indication of slightly slower division compared to cells primed with gag p24 alone.

Importantly, an analysis of the T cell response to the conjugate LTB-gag p24 revealed a significant shift of the peaks to the left with an increase in the number of cells in generations 4 and 5 (Figure 3B), an indication of an increase in the T cell division rate. It is concluded that conjugation of HIV gag p24 to LTB confers enhanced T cell division rate in response to gag p24.

### Gag p24 does not adversely affect MHC class II-restricted T cell division-Addition of LTB enhances T cell division in response to an unrelated antigen (OVA)

Previous reports [27,28] have indicated that HIV-derived proteins, including gag p24, adversely affect MHC class I antigen presentation to an unrelated antigen. It has been suggested that inhibition of the immune response allows viruses including HIV to escape the immune system. However, no data is available whether HIV gag p24 alters antigen presentation by the MHC class II pathway. Inhibition of MHC class II antigen presentation would have an adverse effect on the stimulation of an appropriate immune response to a multivalent vaccine. Therefore, an experiment was set up to examine whether gag p24 adversely affects T cell division of OVA-specific DO.11.10. Additionally, the effects of adding LTB to these cultures were investigated.

Splenic cells were isolated and used as a source of APCs (~65%). The cells were incubated in the absence of antigen, with OVA, OVA+gag p24, OVA+gag p24+LTB; and OVA+LTB, for 6 hrs at 37°C to allow uptake of the antigens. Gag p24 was used at increasing concentrations of 10, 20 and 40 μg/ml. OVA and LTB were added at 0.5 mg/ml and 30 μg/ml, respectively [29]. DO.11.10 T cells were pre-labeled with a predetermined optimum concentration of CFSE and added to the splenic cultures. The cells were then incubated for 3 days and analyzed by flow cytometry.

**Figure 4:**
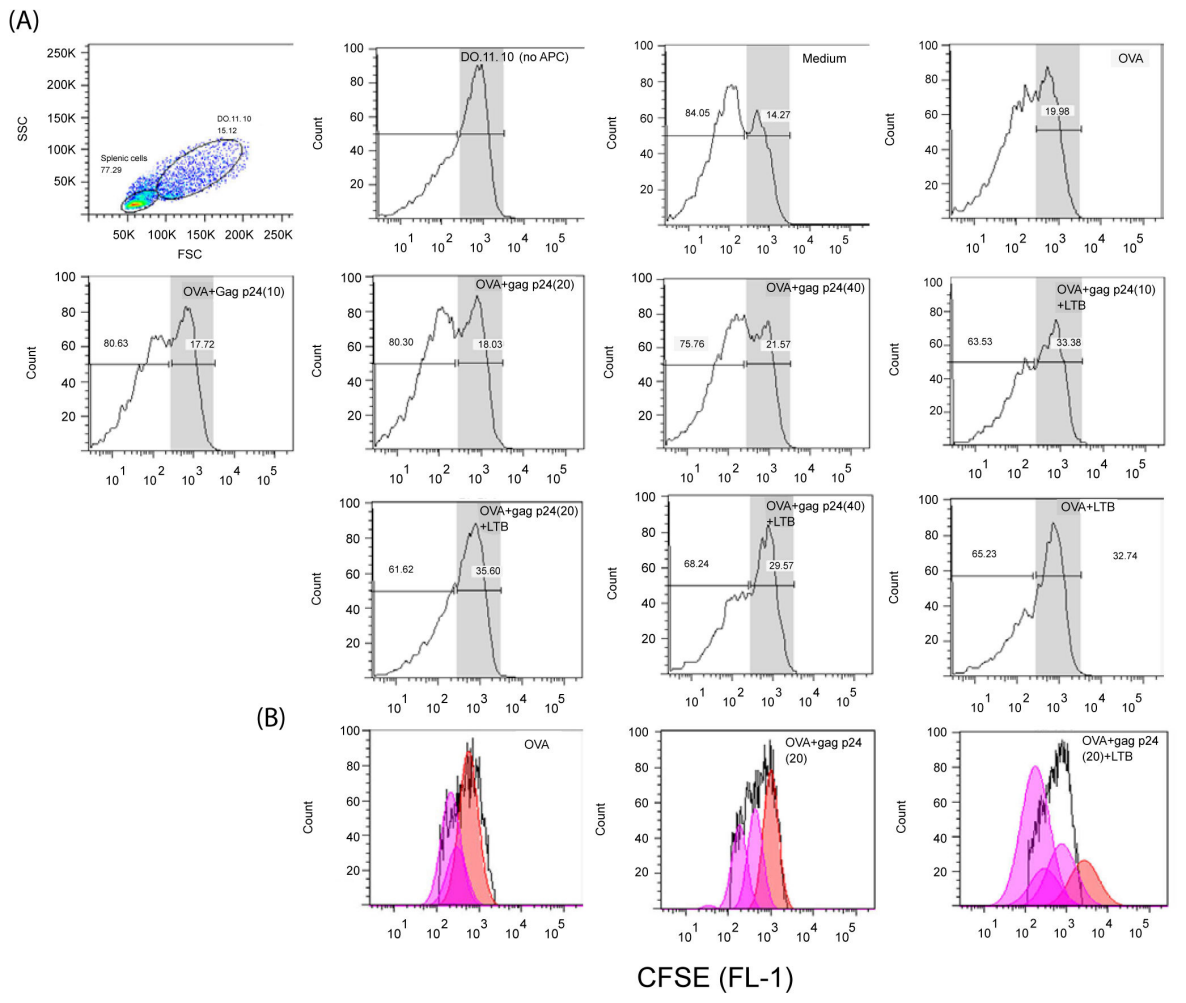
Effects of Gag p24 and LTB on antigen presentation of an unrelated antigen, OVA Splenic cells were isolated from normal mice and incubated in the absence of antigen (medium) or with OVA (0.5 mg/ml); OVA (0.5 mg/ml)+gag p24 (10, 20 and 40 μg/ml); OVA (0.5 mg/ml)+gag p24 (10, 20 and 40 μg/ml)+30 μg/ml LTB; or OVA (0.5 mg/ml)+LTB (30 μg/ml), for 6 hrs at 37°C to allow uptake. DO.11.10 T cells hybridoma were pre-labeled with CFSE and added to the culture above. The cells were then incubated for 3 days and analyzed by flow cytometry. In **(A)**, the dot plot analysis shows 2 populations of cells of lower and higher FSC representing splenic cells and DO.11.10 respectively. Dead cells of very low FSC and high SSC were gated out. CFSE-labeled DO.11.10 cells are also shown in histogram after gating on the population above. The other histograms show number of DO.11.10 cells in the presence of various treatments as indicated. In **(B)**, the histograms show proliferation profile of those DO.11.10 T cells in cultures incubated with OVA, OVA+gag p24 (20 μg/ml) and OVA+gag p24 (20 μg/ml)+LTB.

Flow cytometry analysis of DO.11.10 T cells after culture is shown in Figure 4. Forward scatter (FSC) versus side scatter (SSC) analysis shows 2 separate populations representing splenic cells (lower FSC and lower SCC) and DO.11.10 T cells (higher FSC and SCC). The 2 populations are also shown in the histograms with percentages of each cell population and the type of antigen added to the culture. DO.11.10 T cells are shaded for enhanced viewing.

The analysis shows (Figure 4A) that in the absence of antigens DO.11.10 cells represent approximately 14% of the total cells in culture. Addition of a stimulatory dose of OVA [29] resulted in an increase in the number of DO.11.10 cells to approximately 20%. The addition of an increasing concentration of HIV gag p24 (10 to 40 μg/ml) did not affect the percentages of cells. The latter did not exceed 21%, in cultures treated with 40 μg/ml. Based on these data, HIV gag p24 does not appear to adversely affect the number of DO.11.10 cells.

Interestingly, the addition of LTB to the cultures above significantly increased the number of cells from approximately 14% in medium cultures to a maximum of 35%. That this increase is due to LTB and not to a synergistic effect of both LTB and gag p24 is shown in that the number of DO.11.10 cells in cultures treated with LTB+OVA alone were approximately 33% of total cells.

Because the increase in the DO.11.10 cell number may be due to better maintenance of the cells in culture by LTB rather than a direct effect on cell division, analysis of cell division was performed (Figure 4B). In the presence of OVA, some cell division is evident by the presence of 3 peaks. Addition of gag p24 resulted in a slight shift to the left which may reflect a slight increase in cell division. However, addition of LTB increased the number of peaks and resulted in a greater shift to the left, an indication of a significant effect of the latter on T cell division.

It is concluded that gag p24 does not appear to adversely affect T cell division to an unrelated antigen, OVA. Addition of LTB to these cultures results in enhanced T cell division, a result which is consistent with its ability to effectively deliver antigens to the immune system.

## Discussion

Control of HIV-1 infection by vaccination is likely to involve multiple approaches including immunization with multivalent vaccine wherein antigens are delivered in suitable platforms and by single or preferably prime/boost regimens. Candidate proteins are those involved in HIV attachment and entry into the cell, including gp120 and gp41 as well as core antigens such as gag p24. That these proteins are important in the control of HIV infection is shown by the presence of strong antibody response in HIV-1 infected individuals [16,17] and induction of cell-mediated T cell response that correlates with control of viral load in non-progressors [13–15]. Here we show that conjugation of HIV gag p24 to LTB enhances the *in vivo* T cell response to gag p24. The effects of gag p24 and LTB are further investigated *in vitro* in OVA-specific DO.11.10 cultures. LTB, but not gag p24 significantly increased T cell division.

To obtain HIV gag p24, the protein was purified from a bacterial expression system of BL21 *E. coli* and by using metal affinity columns. The resulting protein is a trimer of ~78 KD which upon reduction migrates as a 26 KD band on SDS-PAGE (Figure 1). Binding of anti-p24 monoclonal antibody to the purified protein confirmed the presence of the immunogen (Figure 1). In the virus, the capsid is formed mostly by hexamers but also pentamers of gag p24 [30].

In blood of HIV-1 infected individuals, gag p24 may be present in various forms including aggregates. The protein is released to the extracellular space following CD4^+^ T cell lysis or after virus destruction by immune cells. Hence, a strong immune response to HIV gag p24 is generated particularly at early stages of HIV infection, which it subsequently wanes or disappears most likely due to the destruction of CD4^+^ T cell help [31]. Availability of CD4^+^ T cell help is essential for CD8^+^ T cell-mediated killing of HIV-infected cells [32]. Hence, enhancement of the overall anti-gag T cell immune response would be required for the control of HIV-1 infection.

In this study, we used LTB as a platform for the delivery of HIV gag p24. Its use offers several advantages. LTB is non-toxic, has a pentameric structure that could deliver antigens in a multivalent form, binds with high affinity to a ubiquitous cell-surface receptor, G_M1_, targets attached antigens to APCs for delivery into MHC class I and class II pathways [20,22], and activates APCs and T cells and stimulate production of their cytokines [19,21]. Thus, the conjugation or genetic fusion of protein antigens to LTB would likely result in enhanced immune responses. Indeed, a number of epitopes and proteins from conventional or infectious agents including HIV have been attached to, plasmid expressed or mixed with LTB and shown to result in enhanced antibody and cellular immune responses. Thus LTB conjugated to OVA peptides induced CD8^+^ T cells that protected against OVA-loaded melanoma [33]. Further, co-injection of plasmids encoding the holotoxin LT or LTB with those expressing HIV gp120 or hepatitis B surface or core antigens adjuvanted the immune response to gp120 [34]. Genetic fusion of the V3 loop of HIV gp120 to LT induced antibodies that neutralized several HIV strains [35]. In this study, a single injection of conjugates of HIV gag p24 and LTB in PBS enhanced the T cell responses to gag p24 compared to gag p24 alone or a mixture of LTB+gag p24. This was evident by the noticeable increase in T cell division rate in response to the conjugate (Figure 3).

A number of mechanisms could explain the better outcome following injection of the HIV gag p24-LTB conjugate. The conjugate would be targeted more efficiently to cell surface G_M1_ on APCs by LTB, a process that significantly enhances stimulation of T cells compared to a nonbinding mutant of LTB or non-conjugated protein [20]. Although both free LTB and LTB conjugated to gag p24 would activate APCs [21], targeting to cell surface G_M1_ by the conjugate would still result in better T cell stimulation presumably due to enhanced uptake and access to MHC compartments [20,22]. Further, LTB may enhance stimulation of cytokines in these cultures and consequently induce maturation of the T cell response. Surprisingly; however, we did not detect IL-4 and IFN-γ in these cultures. It should be restated that the proteins were injected once in PBS. It is very likely that a number of injections would be required to induce maturation of the T cell response to gag p24. Nevertheless, LTB appears to effectively deliver gag p24 to the APC, a necessary step in the priming events of the immune response.

HIV proteins have been reported to inhibit immunogenicity and antigen presentation when co-delivered with other proteins. Thus co-delivery of plasmids encoding for HIV gp120 with plamids encoding OVA inhibited level of expression and antigen presentation of OVA as measured by level of OVA-specific CD8^+^ T cells and secretion of IL-2 [27]. The inhibitory effects of gp120 were attributed to direct effects on antigen APCs through epitopes competition. Moreover, HIV gag p24 was found to inhibit class I-mediated antigen presentation of OVA by altering the composition of the immunoproteasome [28]. Inhibition of the immune response by HIV proteins has been suggested as a virus strategy to escape from the immune system. Because future vaccines against HIV ought to increase the breadth of the immune response, inclusion of several HIV proteins or epitopes is a likely possibility, hence; the necessity to address this issue.

Here we looked at effects of adding gag p24 to DO.11.10 cultures (Figure 4). We found that addition of an increasing concentration of gag p24 up to 40 μg/ml did not decrease the number or cell division rate of DO.11.10 cells. Interestingly, the *in vitro* addition of LTB to these cultures significantly increased the number and rate of division of these cells. There are a number of explanations for the different outcome in our study compared to the studies above. First, while the other studies addressed the inhibitory effects of HIV proteins on class I antigen presentation we looked at consequences of adding HIV gag p24 on the class II-restricted DO.11.10 cells. Second, the T cell response of the hybridomas in the studies above was determined by measuring levels of IL-2 secretion whereas in our study we determined the number and the T cell division rate of OVA-specific DO.11.10 cells. Finally, in one of these studies [28], inhibition of antigen presentation by HIV gag p24 was found when dendritic cells, but not macrophages were used whereas in the current study whole splenic cells were the source of the APCs.

The mechanism by which LTB increased DO.11.10 cell division rate is unclear. LTB may enhance activation of the APCs in culture increasing their ability to take up OVA and resulting in higher level of IL-2. However, we found that LTB did not increase stimulation of DO.11.10 cells when OVA were presented by A20 B cells either in soluble form or when targeted to the B cell receptor [29]. It is possible though that LTB may increase antigen uptake by other splenic APCs.

We conclude that the use of LTB as a platform for the delivery of HIV gag p24 enhances the anti-gag T cell response. The nature of this response remains to be determined. The pentameric structure of LTB and its ease for genetic manipulation allow further to engineer fusions of HIV proteins with a defined structure for inclusion in vaccines as priming agents.
